# Multiple Pulmonary Metastases following Total Removal of a Bilateral Parasagittal Meningioma with Complete Occlusion of the Superior Sagittal Sinus: Report of a Case

**DOI:** 10.1155/2012/121470

**Published:** 2012-07-15

**Authors:** Masataka Nakano, Toshihide Tanaka, Aya Nakamura, Mitsuyoshi Watanabe, Naoki Kato, Takao Arai, Yuzuru Hasegawa, Tadashi Akiba, Hideki Marushima, Yukiko Kanetsuna, Toshiaki Abe

**Affiliations:** ^1^Department of Pathology, Jikei University School of Medicine Kashiwa Hospital, 163-1 Kashiwa-shita, Chiba 277-8567, Kashiwa, Japan; ^2^Department of Neurosurgery, Jikei University School of Medicine Kashiwa Hospital, 163-1 Kashiwa-shita, Chiba 277-8567, Kashiwa, Japan; ^3^Department of Thoracic Surgery, Jikei University School of Medicine Kashiwa Hospital, 163-1 Kashiwa-shita, Chiba 277-8567, Kashiwa, Japan; ^4^Department of Neurosurgery, Jikei University School of Medicine, Chiba 277-8567, Kashiwa, Japan

## Abstract

Pulmonary metastases of benign meningiomas are extremely rare. The case of a 34-year-old man with bilateral parasagittal meningioma who developed pulmonary metastases is described. The meningioma was an enormous hypervascular tumor with invasion of the superior sagittal sinus. The tumor was resected completely and histologically diagnosed as transitional meningioma. The Ki-67 labeling index was 5%. Four months after operation, the patient subsequently developed bilateral multiple lung lesions later identified as metastases. The lung lesions were partially removed surgically and histologically diagnosed as meningothelial meningioma WHO grade I. The Ki-67 labeling index was 2%. The histological findings demonstrated that the tumor occupied the arterial lumen and the perivascular space, suggesting that pulmonary tumors might metastasize via the vascular route. The histopathological features and mechanisms of metastasizing meningiomas are reviewed and discussed.

## 1. Introduction

Meningioma is one of the most frequently encountered tumors of the central nervous system; it consists of meningoepithelial cells and generally originates from intracranial meninges. Meningiomas are considered benign lesions that are not locally invasive and are usually cured by surgical resection [1]. Atypical (WHO grade II) and anaplastic (WHO grade III) meningiomas show more aggressive biological behavior, with a high risk of local recurrence and a less favorable prognosis.

However, meningiomas have a potential to become more aggressive and invade the brain and/or calvaria or to metastasize outside the central nervous system. According to previous reports, most meningiomas with pulmonary metastases were histologically atypical (WHO grade II), anaplastic (WHO grade III) or initially benign meningiomas that changed to atypical or anaplastic tumors on recurrence [2, 3]. Metastatic meningioma is quite rare, and the lung is the most frequent site for metastases [4].

There are no definitive criteria to predict local recurrence or metastases in meningiomas. Metastases outside the central nervous system are known to occur rarely even from meningiomas that at first diagnosis are considered grade I tumors.

The patient case was a recurrent parasagittal meningioma that invaded the superior sagittal sinus and eventually metastasized to the lung. Histologically, the intracranial meningioma was transitional type, whereas the pulmonary meningiomas were meningothelial meningiomas. The proliferative potential of the intracranial and pulmonary meningiomas was evaluated immunohistochemically by Ki-67 labeling (5% and 2%, resp.).

In view of the slow-growing nature of these metastases and their good prognosis after resection, surgery is the treatment of choice [1].

## 2. Case Report

### 2.1. History of the Intracranial Meningioma

**Figure 1:**
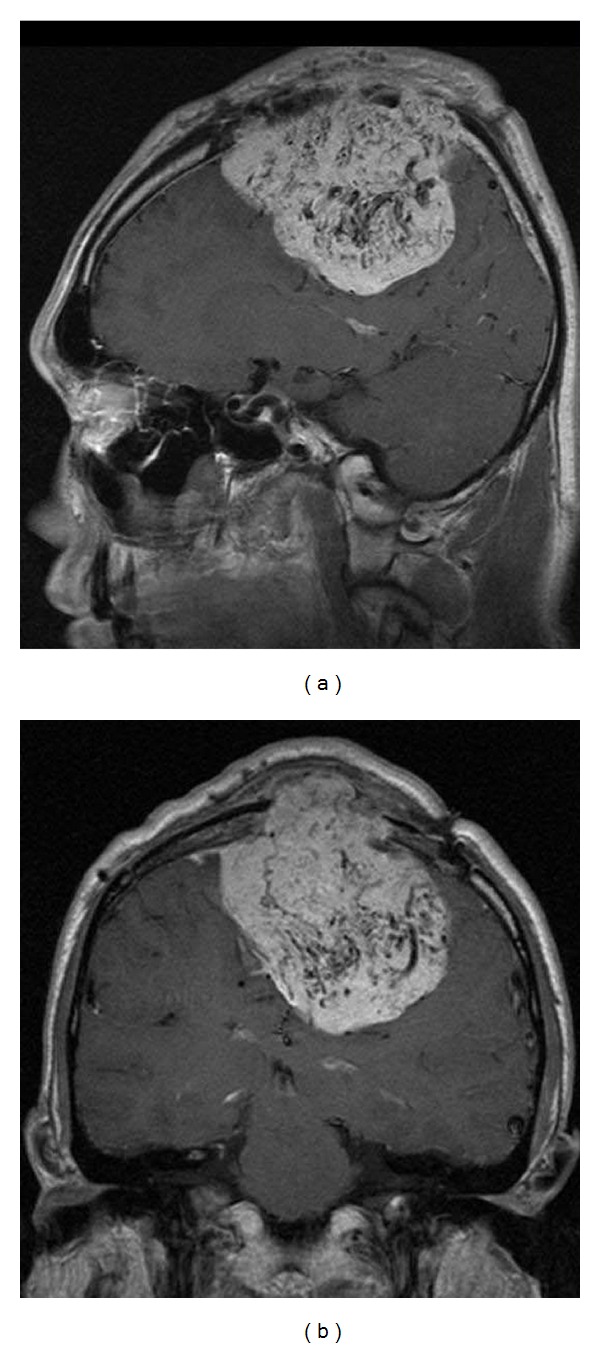
Preoperative sagittal (a) and coronal (b) T1-weighted magnetic resonance (MR) images with contrast medium showing the huge parasagittal meningioma. Note the dense flow void networks in the tumor.

**Figure 2:**
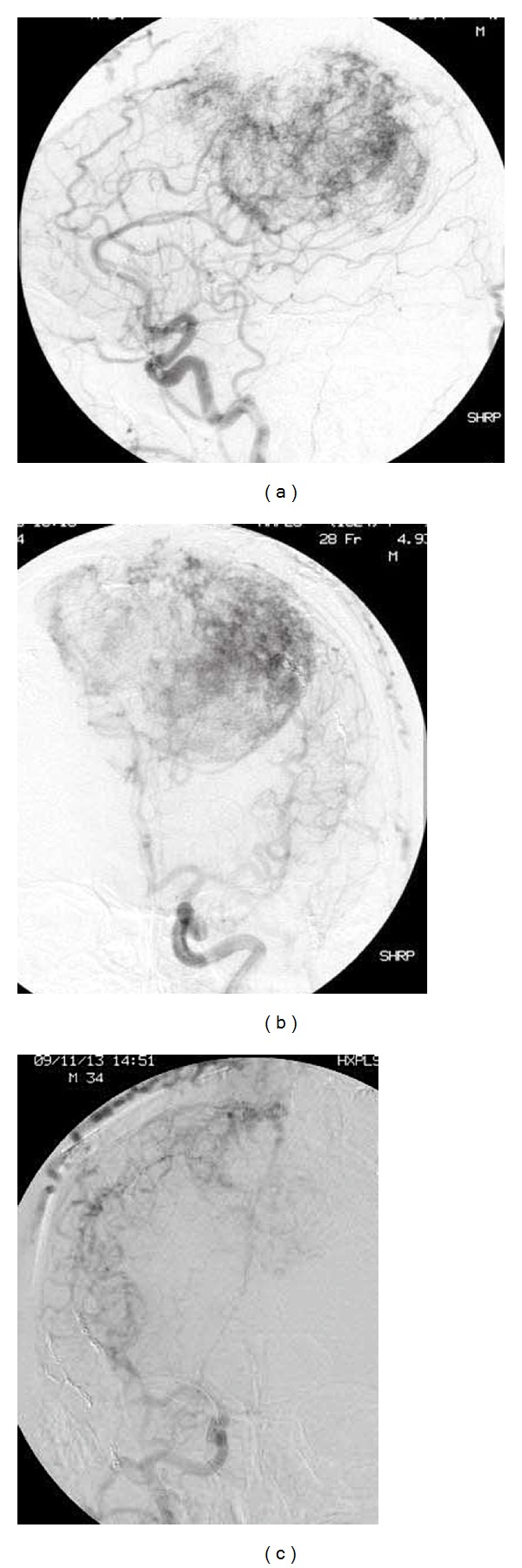
(a) Left carotid angiogram (CAG) revealing the microvascular networks in the tumor. Lateral (a) and anterior-posterior (b) views of CAG demonstrating that the tumor is fed by the anterior falcian, precentral, and central arteries. External carotid angiogram (c) demonstrating that the tumor is fed by the middle meningeal and superficial temporal arteries.

A 34-year-old man who had previously undergone craniotomy a total of three times in 2005 and 2006 presented with a progressive gait disturbance and was referred to our institution for further treatment. He presented with paraplegia involving bilateral lower extremities and a memory disturbance. Computed tomography (CT) revealed a huge isodense mass, approximately 8 cm in diameter, with strong perifocal edema involving the falx and superior sagittal sinus (SSS). The tumor invaded the calvarium and scalp, with compression of bilateral frontal lobes. Magnetic resonance imaging (MRI) showed the mass as isointensity on T1-weighted imaging and heterogeneously intense on T2-weighted imaging, with heterogeneous enhancement with gadolinium (Figures 1(a) and 1(b)). Intratumoral flow voids were diffusely distributed. Cerebral angiography demonstrated that the tumor was fed by the anterior falcian, precentral, central, and middle meningeal arteries, as well as a thick branch that originated from the tentorial artery (Figures 2(a), 2(b), and 2(c)). The SSS was not visible, and the cortical veins as draining veins were dilated and drained into the sphenoparietal sinus and the superior petrosal sinus. The fourth craniotomy was carried out in 2009.

### 2.2. Operative Findings of the Intracranial Meningioma

**Figure 3:**
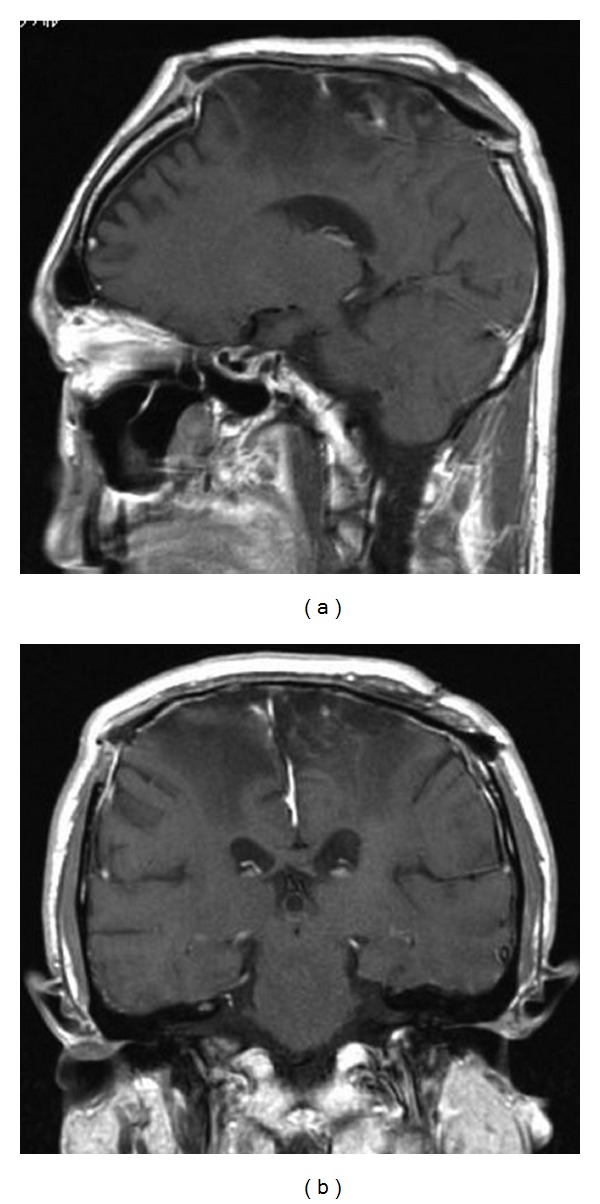
Postoperative sagittal (a) and coronal (b) T1-weighted MR images with contrast medium revealing complete removal of the tumor without recurrence.

The tumor was highly vascular, composed of elastic-hard, fibrous, grayish-white tumor and soft reddish components that invaded the calvarium. The tumor adhered tightly to the cerebral cortex with involvement of the falx and SSS, and there was arterial bleeding from the falx. Dense vascular networks were cauterized, the SSS was completely obliterated, and the tumor was dissected from the edematous cortex and then totally removed. Blood loss was 6500 mL. Postoperatively, the patient had paraparesis and was transferred for extensive rehabilitation. Postoperative MR images showed complete removal of the tumor without recurrence (Figures 3(a) and 3(b)).

### 2.3. History of the Pulmonary Meningioma

**Figure 4:**
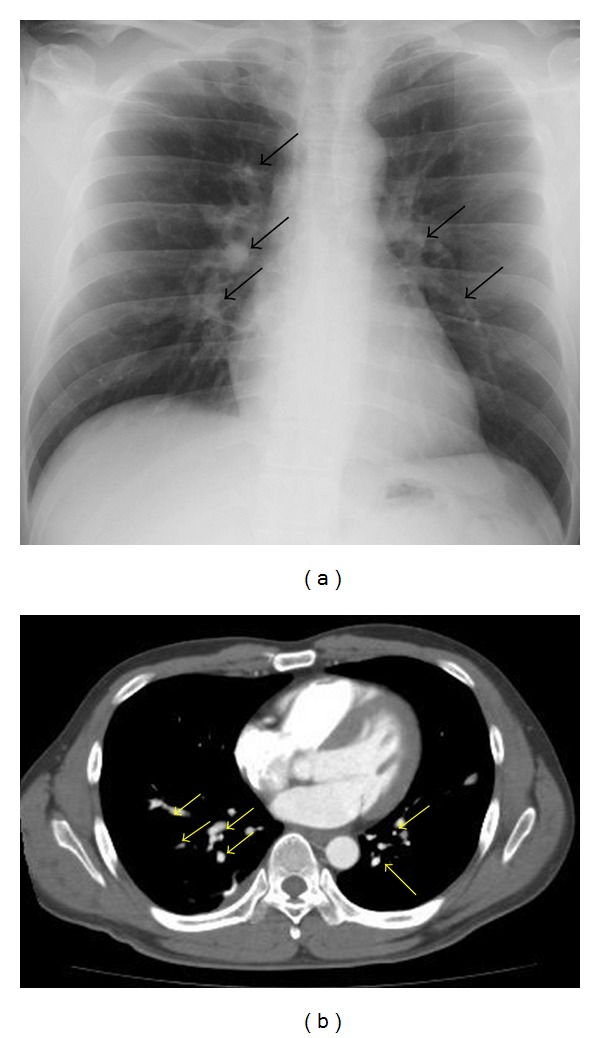
Chest radiograph (a) and computed tomography (CT) (b) reveal multiple tumors in both lobes. Arrows represent meningioma metastases.

**Figure 5:**
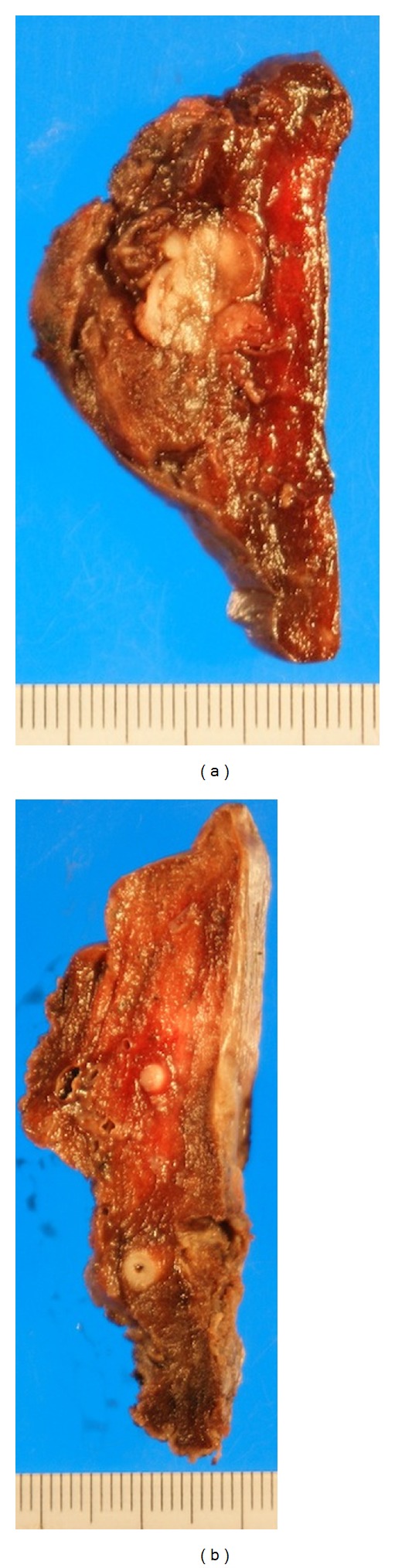
Gross appearance of the pulmonary meningioma in right S3 (a) and S6 (b). The excised tumor consists of a yellow-white portion extending into the lung parenchyma.

Two months after the last operation, X-rays and CT scans of the chest demonstrated well-circumscribed, multinodular tumors in bilateral lungs (Figures 4(a) and 4(b)). The right basal posterior segment (S10) of the lung was partially resected thoracoscopically and initially diagnosed as “meningioma.” Two months after biopsy, the posterior segment (S2) and the superior segment (S6) were resected. The specimen showed two nodules in the upper lung (one in each of S2 and S3) and five nodules in the lower lobe (S6). These seven nodules measured 1-2 cm. Macroscopically, the multiple nodules in the lung had well-defined borders, whitish color, and firm consistency (Figures 5(a) and 5(b)). The patient's postoperative course was uneventful.

### 2.4. Histological Findings

**Intracranial Meningioma**

**Figure 6:**
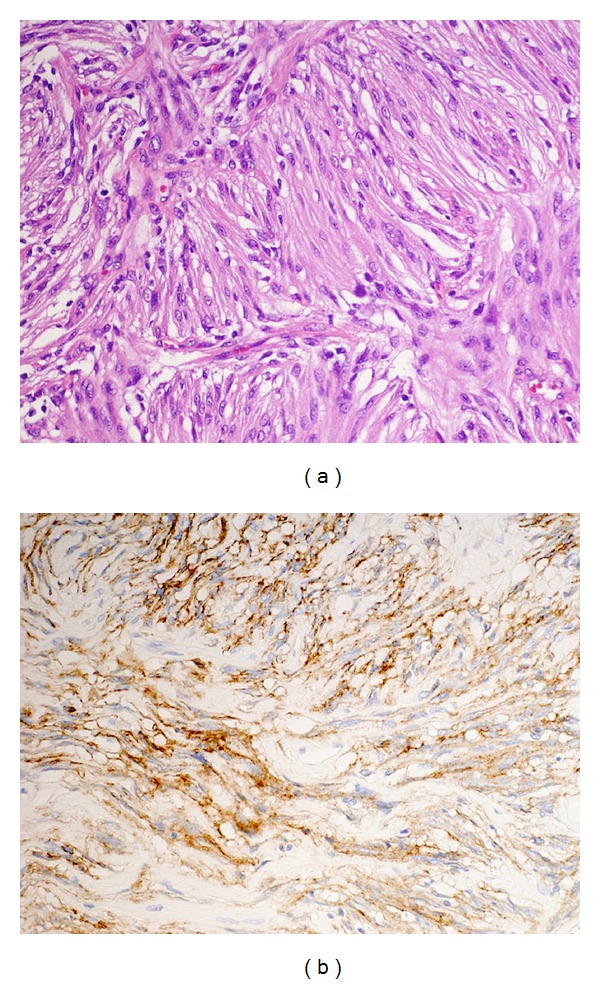
Photomicrographs revealing a highly cellular tumor consisting of oval and spindle cells and delicate fibroconnective tissue in parallel bundles (a) (hematoxylin and eosin stain, original magnification: ×200). Tumor cells are positive for epithelial membrane antigen (EMA) (b: ×200).

The recurrent intracranial tumor showed spindle-shaped cells with atypical nuclei and high cellularity, together with marked collagen deposition and whorl formation (Figure 6(a)). Lobular and fascicular arrangements were present. Approximately 5% of the tumor cells were Ki-67/MIB-1 positive. No mitotic figures were found. The tumor was immunoreactive for vimentin and epithelial membrane antigen (EMA), confirming the diagnosis of transitional meningioma (Figure 6(b)). These morphological and immunohistochemical findings were consistent with a WHO grade I meningioma.

**Pulmonary Meningioma**

**Figure 7:**
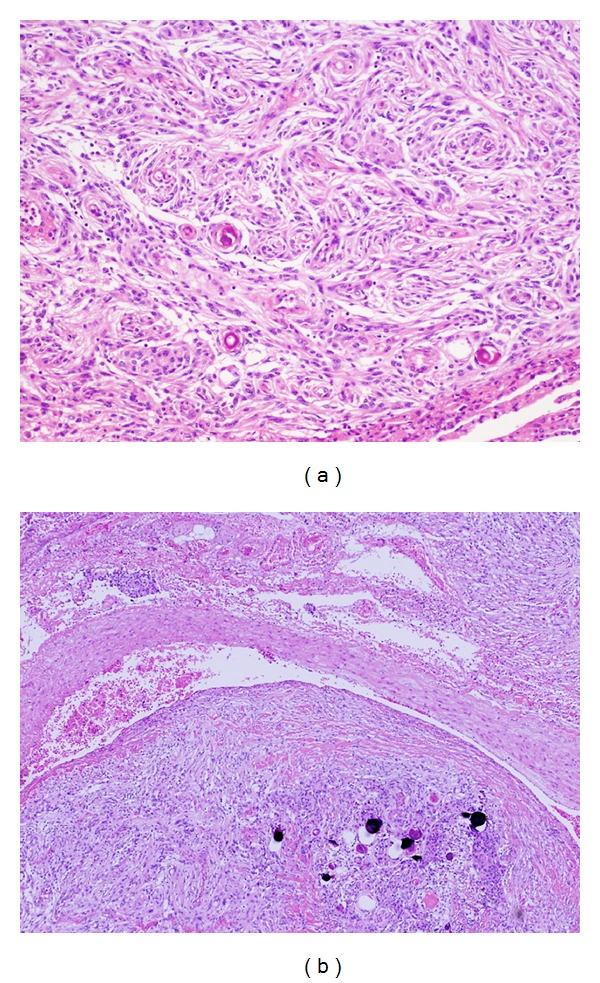
Photomicrograph reveals spindle-shaped cells organized into whorl formation of the meningioma. The tumor cells are seen in the interstitial tissue (a) and to be intravascular (b).

Histopathological examination showed spindle meningothelial cells arranged in fascicules with psammoma bodies infiltrating the lung (Figure 7(a)). As shown in Figure 7(b), the tumor containing psammoma bodies also occupied the arterial lumen, as well as the perivascular space, suggesting that the pulmonary meningioma metastasized via the vascular route. There were no signs of cellular pleomorphism, increased cell density, prominent nucleoli, or necrosis. Immunohistochemical examination parameters of the lung nodule matched those of the intracranial tumor. This pulmonary tumor was diagnosed as meningothelial meningioma. The Ki-67/MIB-1 labeling index was about 2%. Mitoses were less than 2 per 10 high-power fields.

These histological and immunohistochemical findings together with the clinical history of a previous intracranial meningioma were suggestive of metastatic meningioma WHO grade I.

## 3. Discussion

Meningiomas (WHO grade I) are usually noninvasive tumors and do not metastasize and, hence, are perceived as benign tumor [1]. Distant metastases from benign meningiomas are extremely rare and almost all of the reported cases were associated with large intracranial tumors [5]. About 0.1% of meningiomas metastasize, most commonly to the lung (61%), followed by liver, lymph node, bone, pleura, and mediastinum [6–10]. The male-to-female ratio of meningioma is 10 : 7, despite the fact that meningiomas are more common in females [11]. The discovery of metastasis often occurs after recurrence of the primary tumor. The interval from the cranial surgery for the primary tumor to discovery of metastasis ranges from 4 months to 15 years [3, 12–14]. The location of meningioma metastases depends on the route of dissemination. Most spread is hematogenous, especially in tumors that invade the dural sinuses. Blood-borne passage of tumor cells through venous channels is the most likely mechanism for distal spread, since metastases are associated with prior surgery or invasion of the venous system [15, 16]. A second route of dissemination is through the cerebrospinal fluid, leading to tumor within the neuroaxis [17]. It has been theorized that surgical manipulation releases tumor from its normally cohesive state into the bloodstream or cerebrospinal fluid.

In the present case, a huge parasagittal meningioma invaded the superior sagittal sinus and metastasized to the lung following surgical removal. This recurrent tumor was accompanied with dense vasculature and occlusion of the superior sagittal sinus. As for histological malignancy, metastasizing meningiomas are usually histologically benign. Characteristically, meningiomas can be locally invasive of the dura, dural sinuses, adjacent bones, and soft tissues.

There are no clear criteria to identify the subset of aggressive tumors that recur locally or metastasize. The literature suggests that previous craniotomy, venous sinus invasion, local recurrence, papillary morphology, and histological malignancy may be risk factors for systemic spread [18, 19]. The cellular proliferative marker Ki-67 may also be useful for evaluating the potential of meningiomas to recur and/or metastasize [7, 18].

On the other hand, others have described that such locally aggressive behavior is not necessarly indicative of malignancy, and it is usually associated with benign cellular features [10, 11, 20]. A high rate of cellular proliferation is not essential for extracranial metastases, and an individual meningioma of virtually any of the histologically benign meningiomas may metastasize [1, 7, 9, 18, 21]. Unfortunately, the clinical behavior of meningiomas does not always correlate with the histological features. Despite the presence of histologically malignant features in up to 9% of meningiomas, only about 0.1% show metastases  [7, 22]. In the present case, the histological features of both intracranial and metastatic pulmonary meningioma were WHO grade I meningioma: they lacked atypical features, had a relatively low mitotic rate of less than 2 per 10 high-power fields, and the intracranial and pulmonary tumors had low Ki-67 labeling index values (5% and 2%, resp.).

The differential diagnosis of benign metastasizing meningioma with pulmonary meningioma or minute pulmonary meningothelial-like nodules is occasionally difficult. The histological features of primary pulmonary meningioma are round, sharply defined pulmonary nodules. The nuclei are round, oval, or fusiform and eccentrically located in an eosinophilic cytoplasm. Because the cell borders are often not recognizable, the tumor cells are arranged partially in sheets or whorls. Certain features are not recognized, such as the formation of psammoma bodies. There is no cellular pleomorphism or mitosis. These characteristics are the same as those of certain cranial meningiomas [12, 13]. In a few areas, the margin of the tumor is not well circumscribed, instead of showing looser areas in which tumor cells appear to be spinning off of blood vessel cells [13].

Primary pulmonary meningioma usually presents as a solitary pulmonary nodule. Only one case of multiple pulmonary meningiomas has been reported. However, the largest tumor was 1.5 cm in diameter, and the tumors of this case did not involve any lung blood vessels [23]. In the present case, though the largest node was 2.0 cm in diameter, there was lung blood vessel involvement in the metastatic tumor.

Metastatic meningiomas usually display histologic clues that help in addressing the diagnosis, such as their multicentricity and blood vessel involvement as a distance from the larger nodules [12, 19]. Minute pulmonary meningothelial-like nodules are histologically identical to cranial meningiomas and pulmonary meningiomas [12, 13, 19]. However, minute pulmonary meningothelial-like nodules are usually multiple but occur in the vicinity of veins in the interstitium. In addition, minute pulmonary meningothelial-like nodules are usually smaller than 3 mm in diameter and radiologically undetectable [24–28]. The presence of multiple pulmonary nodules that are larger than 3 mm suggests a diagnosis of metastatic meningiomas from the central nervous system. Moreover, the present case showed evidence of lung blood vessel infiltration as shown in Figure 7(b).

The other differential diagnoses of meningiomas include hemangiopericytoma and solitary fibrous tumor. Hemangiopericytomas have the classical staghorn pattern at low magnification and a distinctive turbulent pattern at higher magnifications [29, 30]. Solitary fibrous tumor is composed of spindle cells disposed in fascicles between prominent, eosinophilic bands of collagen. Hemangiopericytoma and solitary fibrous tumor show strong immunoreactivity for vimentin and CD34, but not EMA [19, 29, 30]. Moreover, since hemangiopericytomas of the meninges metastasize frequently, the differential diagnosis is important [7]. In the present case, hemangiopericytoma and solitary fibrous tumor were ruled out by the morphological and immunohistochemical characteristics.

Benign meningiomas commonly invade into the dural sinuses and thus have access to the vascular system. However, dural sinus invasion is not predictive of hematogenous dissemination. It is impossible to disregard the mechanical spread of cells during surgery or as a result of vascular wall invasion. On the other hand, different authors have drawn attention to the low incidence of metastasis associated with surgical management, as well as the frequent involvement of the venous sinuses in meningiomas, because some cases were not operated on before metastases were discovered [10, 11, 26]. The rarity of extracranial metastases in meningiomas may be due to the strong cohesiveness of these tumor cells, as well as the possibility that the extracranial organs may not supply a “fertile soil” for these tumor cells [10, 20, 25]. Pulmonary metastatic meningiomas represent a typical example of benign tumors that may implant into the lungs. The tumors are definitely considered benign, but unusual secondary localization cases are reported [11].

In the present case, there has been absolutely no recurrence and no metastases clinically and radiologically to date. In view of the slow-growing nature of metastatic meningiomas and their good prognosis after resection, surgery is the treatment of choice.
